# School practices to promote social distancing in K-12 schools: review of influenza pandemic policies and practices

**DOI:** 10.1186/s12889-018-5302-3

**Published:** 2018-03-27

**Authors:** Lori Uscher-Pines, Heather L. Schwartz, Faruque Ahmed, Yenlik Zheteyeva, Erika Meza, Garrett Baker, Amra Uzicanin

**Affiliations:** 1 RAND Corporation, Arlington, VA USA; 2 Division of Global Migration and Quarantine, National Center for Emerging and Zoonotic Infectious Diseases, CDC, Atlanta, GA USA

**Keywords:** Pandemic influenza, School planning, Social distancing

## Abstract

**Background:**

During an evolving influenza pandemic, community mitigation strategies, such as social distancing, can slow down virus transmission in schools and surrounding communities. To date, research on school practices to promote social distancing in primary and secondary schools has focused on prolonged school closure, with little attention paid to the identification and feasibility of other more sustainable interventions. To develop a list and typology of school practices that have been proposed and/or implemented in an influenza pandemic and to uncover any barriers identified, lessons learned from their use, and documented impacts.

**Methods:**

We conducted a review of the peer-reviewed and grey literature on social distancing interventions in schools other than school closure. We also collected state government guidance documents directed to local education agencies or schools to assess state policies regarding social distancing. We collected standardized information from each document using an abstraction form and generated descriptive statistics on common plan elements.

**Results:**

The document review revealed limited literature on school practices to promote social distancing, as well as limited incorporation of school practices to promote social distancing into state government guidance documents. Among the 38 states that had guidance documents that met inclusion criteria, fewer than half (42%) mentioned a single school practice to promote social distancing, and none provided any substantive detail about the policies or practices needed to enact them. The most frequently identified school practices were cancelling or postponing after-school activities, canceling classes or activities with a high rate of mixing/contact that occur within the school day, and reducing mixing during transport.

**Conclusion:**

Little information is available to schools to develop policies and procedures on social distancing. Additional research and guidance are needed to assess the feasibility and effectiveness of school practices to promote social distancing.

## Background

During communicable disease outbreaks such as pandemic influenza, social distancing interventions that increase the space between people and decrease the frequency of contacts can play an important role in emergency response [1, 2]. Influenza pandemics typically have multiple waves and can last for months [3, 4]. The most recent pandemic in the United States was the 2009–2010 H1N1influenza pandemic, which disproportionately impacted children and young adults [5, 6].

During an evolving influenza pandemic, community mitigation strategies, such as social distancing, can slow down virus transmission in schools and surrounding communities, helping to relieve pressure on over-burdened healthcare and public health systems and buy time for pandemic vaccine production and distribution [7]. Because schools are socially dense environments where students congregate for many hours of the day, schools can fuel community-wide disease transmission [8, 9]. It follows that school practices that promote social distancing could potentially protect large numbers of vulnerable children, as well as limit secondary transmission to adults within their households and communities.

Despite the potential impact of school practices on disease transmission, research on school practices to promote social distancing in primary and secondary schools has focused on prolonged school closure [10–12], with little attention paid to the identification and feasibility of other more sustainable interventions that are less costly for society. Although many states and grant programs require school districts and individual schools to plan for infectious disease outbreaks, such as pandemic influenza, there is a paucity of published research on the content of these planning documents. Thus, pandemic influenza guidance documents and plans produced by state governments are a potential source of information on school practices to promote social distancing, as well as practical and policy barriers to the adoption of those practices.

To fully characterize and identify gaps in the literature on school practices to promote social distancing in a communicable disease outbreak, we reviewed both peer-reviewed and grey literature on social distancing interventions in schools other than school closure. We also collected and catalogued state government planning documents to assess state policies regarding social distancing interventions. Our aims were to develop a list and typology of school practices that have been proposed and/or implemented, summarize any barriers identified and lessons learned from their use, and summarize documented impacts. Such a typology can be of use to state and local education agencies as they consider their options for emergency planning.

## Methods

### Literature review

From September–October 2016, the lead author and research assistant independently conducted searches of 10 education, public health, and general library catalogue databases. Multiple databases (e.g., EBSCO Information Services, WorldCat, New York Academy of Medicine) cover both peer-reviewed and grey literature sources, including reports, newspaper, newsletter, and magazine articles. We restricted our search to articles published after 2000.

Within each database, either the lead author or research assistant first conducted a broad search using the search terms “school and (pandemic or influenza).” In instances where that search strategy yielded fewer than 1000 results, we reviewed all abstracts and did not run additional searches. In contrast, when more than 1000 results were returned, we conducted two additional, narrower searches. We first re-ran “school and (pandemic or influenza),” and required that these terms appear in the title and/or abstract. We then also ran the search terms “school AND (influenza or pandemic) AND (social distancing or practices or strategies or measures or interventions).” Table 1 shows the databases searched, final search terms, and results.

**Table 1:** Literature Search Strategy, 2000–2016

Database	Search Terms	Results
EBSCO (Academic Search Premiere)	School (abstract) AND (pandemic or influenza) (abstract)	998
Education abstracts	(school) and (pandemic or influenza)	214
Eric	(school) and (pandemic or influenza)	143
Google Scholar	(school and (influenza or pandemic))	200^a^
JSTOR	((ab:(school) AND ab:(influenza or pandemic)) AND ((social distancing or practices or interventions or strategies or measures)))	66
Psych INFO	(schools and pandemic or influenza and (social distancing or practices or measures or interventions or strategies)	106
PubMed	(schools[Title/Abstract]) AND ((social distancing)[Title/Abstract] OR influenza[Title/Abstract] OR pandemic[Title/Abstract])	480
Scopus	School and (influenza or pandemic) (AB)	610
Social Sciences Abstracts	school and (pandemic or influenza) (Title/Abstract)	104
Sociological Abstracts	school and (pandemic or influenza) (Title/Abstract)	40
Web of Science	TITLE: (school) *AND* TOPIC: ((pandemic or influenza) and (practice or strategy or measure or intervention or social distancing))	215
WorldCat	school and (influenza or pandemic) and (strategies or measures or interventions or practices or social distancing)	891

Notes: ^a^Only first 200 results reviewed

Both the lead author and research assistant reviewed the same set of approximately 2000 unique abstracts that were identified through these database searches, as well as the reference lists of relevant articles, selecting 46 articles for full text review. Abstracts were excluded if they were: (1) not related to K-12 school practices or policies to promote social distancing in an infectious disease outbreak; (2) focused on schools other than K-12 (e.g., university planning for pandemic influenza); (3) published in a language other than English; or (4) only focused on sustained school closure rather than other potential school practices. The majority of abstracts were excluded because they were not related to K-12 school practices or policies to promote social distancing in an infectious disease outbreak (e.g., abstract focused on routine hygiene practices in schools).

Of the 46 articles that were selected for full text review, 30 did not mention a single (non-school closure) strategy, leaving 16 for our review.

To obtain relevant information from each reviewed article in a standardized manner, we developed an abstraction form. The form included the following items: article abstract, study design, study location/population, school practices identified, barriers to implementation, impact of school practices, and notes/observation. A researcher or research assistant collected data on each article using that form.

### State government planning document review

We first conducted Google searches to identify state-level documents from 50 states and the District of Columbia to support public school planning. We sought to identify one document per state that provided guidance to its local education agencies (LEA) or schools. We excluded general pandemic influenza preparedness plans that were not directed to local education agencies or schools. To locate these documents, we conducted online searches that had various combinations of the following terms: [name of state], school, education, pandemic influenza, infectious disease, communicable disease, emergency response, guidance, and plan. Our goal was to identify pandemic-specific documents, either stand-alone documents or chapters or annexes in a larger emergency plan. However, when we could not identify a document specific to pandemic influenza, we then searched for materials related to infectious disease or emergency planning more broadly. In instances where we identified more than one guidance document within a state that met inclusion criteria, we selected the document that was published most recently.

Each plan/planning document was reviewed by a researcher or research assistant, and the following information was extracted: state name, type of plan (pandemic, infectious disease, emergency plan), author of plan, year plan was written, whether hygiene practices were discussed, whether school closure was discussed, whether distance learning was discussed, whether school practices to promote social distancing were discussed (and if yes, what types), and any included details about barriers, facilitators, or considerations for social distancing. RAND’s Institutional Review Board approved this study, determining that it did not involve human subjects and informed consent did not apply.

## Results

### Literature review

Of the 16 articles that met all inclusion criteria, 6 (38%) presented the results of agent-based simulation studies/modeling, and 5 (31%) presented the results of surveys regarding school practices during the 2009 H1N1 pandemic. The other five articles were a combination of commentaries, magazine or newsletter articles, and miscellaneous research articles.

Included articles identified more than a dozen different types of school practices to promote social distancing, many of which were slight variations on one another. Table 2 shows these practices as they were initially presented.

**Table 2:** Articles Included in Literature Review, 2000–2016

Article	Article Type	Location	School Practice(s)
Agent-Based Simulation Model			
Ridenhour [20]	Peer-reviewed	N/A	Hall restriction: Defined walking area between classrooms, lunchroom, and schoolyard (e.g., right-hand side of any hall); classroom restriction: Must remain seated while in class; schoolyard restriction: Must stay in a randomly specified schoolyard area (may or may not be classroom-specific); lunchroom restriction: Must only eat with classmates; different schedules/each classroom follows one of three schedules (current schedule, a shift of 45 min, a shift of 90 min)
Adalja [31]	Peer-reviewed	N/A	Segregating small clusters of children to different parts of the room^a^; lunch in classrooms/no congregating for lunch^a^; cancel gym class^a^
Fumanelli [32]	Peer-reviewed	N/A	Class or grade closure (in contrast to full school closure)^a^
Gemmetto [33]	Peer-reviewed	N/A	Class closure; grade closure
Lofgren [34]	Peer-reviewed	N/A	Closure of playground/common areas
Cooley [35]	Peer-reviewed	N/A	Shorter school week: 4 days instead of 5
Commentary/ Newsletter/Magazine Article			
McGiboney [13]	Commentary	DeKalb County, Georgia	Cancel fieldtrips; cancel afterschool activities; distance learning (e.g., cable channel lesson plans, study packets, online lessons)
Education Digest [36]	Newsletter/ Magazine	National	Move desks apart; cancel classes that bring together children from different classrooms
Ash [14]	Newsletter/ Magazine	National	Distance learning
Surveys of Practices during 2009 H1N1 Pandemic			
Miller [16]	Peer-reviewed	Pennsylvania	Cancel activities
Nasrullah [17]	Peer-reviewed	Georgia	Cancel or postpone activities
Rebmann [18]	Peer-reviewed	26 states	Discourage face-to-face meetings in schools
Shi [15]	Peer-reviewed	Michigan	Rearrange classroom to keep students further apart Cancel or postpone school activities such as field trips, school performances, practices, and games
Dooyema [19]	Peer-reviewed	Michigan	Move desks apart; cancel or postpone fieldtrips, performances, practices, after-school programs; divide classes into smaller groups; hold class outdoors (0%); move classes into larger spaces; crowd-reducing methods of transportation (e.g., no busing)
Epidemiologic Study			
Stehlé [37]	Peer-reviewed	France^a,b^	Class closure^a^, altered schedules to prevent mixing^a^
Sugisaki [38]	Peer-reviewed	Japan^c^	Grade closure; class closure; later start to school day; cancel activities

^a^ Interventions not tested as described. Discussed in discussion section

^b^ Data collection on face-to-face interactions

^c^ Analysis of school closure data

For ease of interpretation, we created categories of practices and calculated how often they were mentioned across articles (Table 3). The practices identified most frequently included canceling or postponing after-school activities (*n* = 6, 38%), increasing space among students during in-person instruction (*n* = 5, 31%), and canceling classes or activities with a high rate of mixing/contact that occur within the school day (*n* = 5, 31%). While canceling or postponing activities was mentioned frequently, it is unclear whether the intent of this practice, as implemented during the 2009–2010 H1N1 pandemic, was to reduce contact among students or a logical response to high rates of absenteeism or community concern.

**Table 3:** Most Common Types of School Practices Discussed in Literature to Create Physical Distance Among Students Enrolled in Brick-and-Mortar Public Schools, 2000–2016

Category	Examples	# (%) (*n* = 16 articles)
Cancelling or postponing after school activities	Cancel performances, sports practices, or games	6 (38%)
Increasing space among students during in-person instruction	Move class outdoors; re-arrange desks to increase space; divide classes into smaller groups; require that students remain seated while in class	5 (31%)
Canceling classes or activities that occur within the school day with a high rate of mixing/contact	Cancel physical education class; cancel field trips; cancel choir	5 (31%)
Partial closure	Closure of one class; closure of one grade	4 (25%)
Reduced schedule	Shorter school week; shorter school day; students come on alternating days	3 (19%)
Suspending use of common areas	Lunch in class rather than in lunch room; no recess	2 (13%)
Segregating students within common areas	Require that students only eat with classmates in lunchroom; require that students stay in assigned section of school yard	1 (6%)
Reducing the load on common areas through altered scheduling	Let classes out at different times so fewer students are in the hall at any one time	1 (6%)
Implementing standard workplace social distancing measures for teachers and other staff	Reduce face to face meetings; cancel staff meetings	1 (6%)
Reducing mixing during transport	Suspend buses; discourage use of public transportation	1 (6%)

Distance learning was mentioned in 2 (13%) articles [13, 14]. Ash et al. explained that distance learning is supported by various technologies such as the Internet, telephone, radio, TV, text messaging via cellphones, e-mail, and podcasts [14]. Furthermore, during the H1N1 pandemic, there were examples of lessons being broadcast on public access television and studies using text messaging for study groups. However, because distance learning is used in combination with other practices such as school closure, partial school closure, or reduced schedule to achieve the social distancing of students enrolled in traditional bricks and mortar K-12 public schools, we do not list distance learning practices in Table 3.

Surveys of school responses to the H1N1 pandemic provided some data on how often schools implemented different practices. No single social distancing practice, aside from school closure, was widely used. The two most common practices were rearranging classrooms to increase the physical distance between students (implemented in 14% of schools across the state of Michigan in one study) and canceling or postponing various school activities (implemented by 5–16% of schools in one study, depending on the activity) [15]. No other practice was implemented by more than 10% of surveyed schools in any given study [16–19]. There were, however, widespread practices to limit disease spread that were not forms of social distancing. For example, schools routinely isolated ill students and promoted hand hygiene and respiratory etiquette during the H1N1 pandemic.

Very few of the articles (*n* = 2) we reviewed discussed barriers to implementing school practices to support social distancing. Ridenhour et al... mentioned the challenge of enforcing restrictions on the movement of students within a school (e.g., hall restriction, lunchroom restriction) [20], while Ash et al touched on the challenges of implementing distance learning in instances where students do not have access to broadband Internet or required hardware, such as laptops [14]. Additionally, young children and their families may not have the necessary technical skills to participate in online instruction, as opposed to the traditional delivery of instruction that occurs in-person.

All of the studies that used agent-based simulation models, and one epidemiologic study, reported that at least one school practice to promote social distancing could reduce contact among students and/or disease transmission (Table 4). Because articles focused on different practices, with the exception of class or grade closure, it is difficult to draw any conclusions about the likely effectiveness of different practices at this phase of research.

**Table 4:** Impact of School Practices on Influenza Transmission, 2000–2016

Article	Type of practice	Impact Summary
Sugisaki [38]	Class closure	Two-day class closure carried out day after 10% absenteeism rate (compared to no class closure or two-day or three-day closures carried out ≥2 days after a 10% absentee rate) is effective for mitigating influenza outbreaks in elementary school; school actions should be conducted at the class level as a basic strategy.
Lofgren [34]	Suspending use of common areas	Closing the playground and other common areas when 5% of students were symptomatic (compared to requiring symptomatic students to leave school) significantly reduced the total number of infected students.
Gemetto [33]	Class and grade closure	While the closure of one class yields a smaller mitigation effect than the closure of the whole elementary school, the closure of the corresponding grade (two classes) leads to a reduction of large outbreak probability and a reduction of epidemic size that are similar to those obtained by closing the entire elementary school.
Fumanelli [32]	Class and grade closure	Reactive gradual (e.g., starting from class-by-class), reactive school-by-school, and county-wide school closure gave comparable outcomes in terms of infection attack rate reduction, peak incidence reduction or peak delay, while national closure of all schools of the country at the same time was not able to reach the same levels of mitigation.
Cooley [35]	Reduced schedule: 3 day weekend	Using a 3-day weekend as an intervention strategy (compared to a 2-day weekend) could be effective at reducing the peak attack rate for mild epidemics similar in severity to the 2009 H1N1 pandemic.
Ridenhour [20]	Classroom restriction, hall restriction, schoolyard restriction, lunchroom restriction, different classroom schedules	Classroom restrictions were the best single intervention at lower infection probabilities. At higher transmission rates, employing staggered classroom schedules is the best single intervention.

### State government planning document review

In our review of planning documents, we found that 38 of 51 (75%) states had published guidance for local education agencies or schools to support planning for pandemic influenza or communicable disease outbreaks in schools (Table 5). Among the guidance documents we identified, 42% (16 of 38) mentioned one or more school practices to promote social distancing. By contrast, almost all guidance documents (*n* = 36, 95%) discussed hand hygiene and/or sanitation, a large majority (*n* = 34, 89%) discussed school closure, and a little less than half (*n* = 16, 42%) discussed distance learning. As noted in Table 5, the plans and guidance were developed from 2002 to 2016. However, 23 (61%) were published on or after 2009, with 7 (18%) in 2009 alone. Pandemic planning was likely accelerated by the H1N1 pandemic that occurred from 2009 to 2010.

**Table 5:** State Government Plans that Include Guidance to Local Education Agencies or Schools on Pandemic Influenza Preparedness

State	Type of plan	Author	Year	Address hygiene	Address school closure	Address distance learning	Listed School Practices to Promote Social Distancing
AL [39]	Pandemic Influenza	AL Department of Education	2014	Yes	Yes	Yes	1) Cancel all extra-curricular activities. 2) Limit or discontinue all meetings, gatherings, field trips, extracurricular activities, etc., until county health department lifts pandemic conditions. 3) Limit or discontinue travel within the school district. 4) Suspend all transportation and work at the bus shop. 5) Limit or discontinue access to vendors and visitors from outside the school district.
AK [40]	Infectious Disease	AK Department of Health and Social Services	2013	Yes	No	No	None listed
AZ [41]	Pandemic Influenza	AZ Department of Education	2009	Yes	Yes	Yes	1) Incorporate flexible work hours and schedules while also utilizing employee spacing techniques to reduce crowding and close proximity (e.g., staggered shifts, telecommuting, teleconference meetings, separate office spaces).
AR [42]	Pandemic Influenza	AR Department of Health	2014	Yes	Yes	No	1) Snow days: simultaneous closure of offices, schools, and other non-essential community activities for a specified period of time.
CA [43]	Pandemic Influenza	CA Department of Education	2014	Yes	Yes	Yes	1) Alternate scheduling 2) Before- and after- school programs closures
CO [44]	Pandemic Influenza	CO Department of Public Health	2009	Yes	Yes	Yes	None listed
CT [45]	Clinical Procedure Guidelines for School Nurses	CT Department of Education	2012	Yes	Yes	Yes	None listed
DC [46]	General Emergency	DC Department of Education	2009	Yes	Yes	Yes	1) Staggered school times 2) Canceling sports events and other mass gatherings 3) Spacing students’ desks three feet apart in small pods or clusters. 4) Discouraging prolonged congregation in hallways, lunch rooms, etc. 5) Staggering bus routes so there are fewer people on each route. 6) Limiting group activities and interaction between classes. 7) Canceling gym class, choir, or other school activities that place individuals in close proximity.
DE [47]	General Emergency	DE Department of Education	2010	Yes	Yes	No	None listed
FL	NG*						
GA [48]	Pandemic Influenza	GA Department of Education	2015	Yes	Yes	Yes	1) Students’ desks be spaced three (3) feet apart 2) Limit group activities and interaction between classes. 3) Cancel or modify gym class, choir or other school activities that place individuals in close proximity. 4) Gatherings of groups larger than normal class size should be cancelled and avoided (e.g. assemblies, recess). 5) Cancel all extra-curricular activities. 6) Prohibit congregation in hall ways and lunchrooms; if possible, serve box lunches in classrooms to avoid gathering of students in the cafeteria; 7) Stagger class changes to avoid large groups of students in the hallway 8) Stagger dismissal for the same reason; 9) Stagger bus routes to reduce the number of students on each bus.
HI	NG*						
ID [49]	Pandemic Influenza	ID Department of Health and Welfare	2006	Yes	Yes	No	None listed
IL [50]	Pandemic Influenza	IL Department of Public Health	2006	Yes	Yes	Yes	None listed
IN [51]	Communicable Disease	IN Department of Health, Epidemiology Resource Center	2015	Yes	No	No	None listed
IA [52]	General Emergency	IA Department of Public Health & Iowa Department of Education	2012	No	No	No	None listed
KS	NG*						
KY	NG*						
LA [53]	Pandemic Influenza	LA Department of Health and Hospitals	2011	Yes	Yes	Yes	None listed
ME	NG*						
MD [54]	Communicable Disease	MD Department of Education; MD Department of Health and Mental Hygiene; MD State School Health Council	2002	Yes	No	No	None listed
MA	NG*						
MI [55]	Pandemic Influenza	MI Department of Community Health	2006	Yes	Yes	Yes	1) Cancel extracurricular activities 2) Modify work practices/schedules
MN	NG*						
MS [56]	Pandemic Influenza	State of MS	2013	Yes	Yes	Yes	None listed
MO [57]	Communicable Disease	MO Department of Health and Senior Services	2011	Yes	Yes	No	None listed
MT [58]	Pandemic Influenza	MT Office of Public Instruction	2007	Yes	Yes	Yes	None listed
NE [59]	General Emergency	NE Department of Health and Human Services	2012	Yes	Yes	Yes	None listed
NV	NG*						
NH [60]	Pandemic Influenza	NH Department of Education, School Health consultant	2008	Yes	Yes	Yes	None listed
NJ [61]	Pandemic Influenza	NJ Department of Health	2015	Yes	Yes	No	None listed
NM [62]	Pandemic Influenza	NM Public Education Department and Department of Health	2007	Yes	Yes	Yes	None listed
NY [63]	Pandemic Influenza	NY Department of Health	2007	Yes	Yes	Yes	1) Cancel any non-academic events (in the case of school closure)
NC [64]	Pandemic Influenza	NC Department of Health and Human Services	UK	No	Yes	No	1) Reduced school activity calendar
ND	NG*						
OH [65]	Pandemic Influenza	OH Department of Education	2009	Yes	Yes	Yes	1) Cancel non-academic events
OK [66]	General Emergency	OK Department of Health	UK	Yes	Yes	Yes	1) Cancel non-academic events
OR [67]	Pandemic Influenza	OR Department of Education	2008	Yes	Yes	Yes	None listed
PA [68]	Pandemic Influenza	PA Department of Health	2009	Yes	Yes	No	None listed
RI	NG*						
SC	NG*						
SD	NG*						
TN [69]	Pandemic Influenza	TN Department of Education and Department of Health	2009	Yes	Yes	Yes	1) Rotating teachers between classrooms while keeping the same group of students in one classroom 2) Canceling classes that bring students together from multiple classrooms 3) Holding classes outdoors 4) Postponing class trips 5) Discouraging use of school buses and public transit 6) Dividing classes into smaller groups, 7) Moving desks farther apart, and 8) Moving classes to larger spaces to allow more space between students
TX [70]	Infectious Disease	TX Association of School Boards	2014	Yes	Yes	Yes	1) Non-essential travel for sports, other competitions, or field trips may be cancelled by a district superintendent or designee.
UT [71]	Pandemic Influenza	UT Department of Health and Utah Office of Education	2006	Yes	Yes	No	1) Spacing students’ desks three (3) feet apart, in small pods or clusters. 2) Discourage prolonged congregation in hallways, lunchrooms, etc. 3) Staggered school times 4) Staggered bus routes, so fewer people are on each route 5) Limit group activities and interaction between classes 6) Cancelling gym classes, choir, or other activities that place individuals in close proximity.
VT [72]	Pandemic Influenza	VVVT Agency of Human Services	2008	No	Yes	Yes	None listed
VA [73]	Pandemic Influenza	VA Department of Education	2008	Yes	Yes	Yes	1) Move desks further apart 2) Maintain space between people when walking in the hallways, 3) other strategies to decrease large numbers of students intermingling such as suspending programs held in the school auditorium, canceling sporting events, and eating lunch in the cafeteria. 4) Rotate teachers instead of students 5) Suspension of activities, including sporting events, arts performances, and classes as determined by the school division superintendent in consultation with the local health department director and community emergency response team. 6) Gatherings of groups larger than normal class size may be limited or suspended during the school day (e.g. assemblies, recess). 7) Bus transportation for students, on and off campus, may be consolidated or suspended. In some instances, staggered bus routes should be considered to decrease the number of students on each bus. 8) Students’ desks be spaced three (3) feet apart. 9) Discourage prolonged congregation in hall ways and lunch rooms. 10) Stagger school schedules 11) Limit group activities and interaction between classes. 12) Cancel gym class, choir or other school activities that place individuals in close proximity 13) Modify school hours/days of operation (i.e., students with last names A-J come to school Monday and Wednesday, students with last names K-Z come to school on Tuesday and Thursday) 14) Stagger school hours (split days or weeks)
WA [74]	Infectious Disease	Office of Superintendent of Public Instruction	2014	Yes	Yes	No	None listed
WV [75]	Pandemic Influenza	WV Department of Education	UK	Yes	Yes	Yes	1) Close non-essential agency functions; 2) Increase telecommuting, flex scheduling and other options; 3) Cancel all public assemblies or after school activities.
WI [76]	Pandemic Influenza	WI Department of Public Instruction	2009	Yes	Yes	Yes	None listed
WY	NG*						

Notes: NG* refers to no guidance to local education agencies or schools that falls within scope; UK: Unknown

Among the 16 guidance documents that mentioned social distancing, the practices identified most frequently included canceling or postponing after-school activities (*n* = 11, 69%), canceling classes or activities with a high rate of mixing/contact that occur within the school day (*n* = 7, 44%), and reducing mixing during transport (*n* = 6, 38%) (Table 6). State-level guidance documents discussed all of the practices identified in the literature and also identified two unique practices not covered elsewhere: instituting homeroom stay (in which students remain in one classroom and teachers rotate in and out) (*n* = 1, 6%) and limiting visitors (*n* = 1, 6%).

**Table 6:** Most Common Types of School Practices Included in State-level Guidance Documents to Create Physical Distance Among Students Enrolled in Brick-and-Mortar Public Schools

Category	Examples	# (%) (n = 16)^b^
Canceling or postponing after school activities	Cancel performances, sports practices, or games	11 (69%)
Canceling classes or activities that occur during the school day with a high rate of mixing/contact	Cancel P.E.; cancel field trips; cancel choir	7 (44%)
Reducing mixing during transport	Suspend buses; discourage use of public transportation	6 (38%)
Increasing space among students during in-person instruction	Move class outdoors; re-arrange desks to increase space; divide classes into smaller groups; require students to remain seated in classroom	5 (31%)
Reduced schedule	Shorter school week; shorter school day; students come on alternating days	4 (25%)
Suspending use of common areas	Lunch in classrooms rather than in lunch room; no recess	4 (25%)
Implementing standard workplace social distancing measures for teachers and other staff	Limit face to face meetings; cancel staff meetings	3 (19%)
Partial Closure	Closure of one class; closure of one grade	2 (13%)
Instituting home room stay^a^	Children remain with one group of children all day and teachers rotate through the room	2 (13%)
Segregating students within common areas	Require that students only eat with classmates in lunchroom; require that students stay in assigned section of school yard	1 (6%)
Reducing density/load in common areas through altered scheduling	Let classes out at different times so fewer students are in the hall at any one time	1 (6%)
Limiting visitors^a^	Do not allow parents or other visitors; restrict vendor access to school	1 (6%)

^a^School practice mentioned only in pandemic plans/guidance and not in the published literature

^b^16 state-level guidance documents mentioned one or more school practices to promote social distancing

In general, state government guidance documents devoted very little space to school practices to promote social distancing and typically included practices among lists of potential response options, without any details regarding barriers, facilitators, or considerations. Only one document discussed barriers or considerations regarding the implementation of school practices to promote social distancing. Connecticut’s guidance document mentioned that any practice that reduces the number of instructional hours will require waivers of instruction requirements.

## Discussion

Our review revealed a very limited literature on school practices to promote social distancing other than school closure and limited incorporation of school practices to promote social distancing into state-level guidance documents. Fewer than half of all states with guidance documents mentioned a single school practice to promote social distancing, and none provided any substantive detail about the policies or practices needed to enact the listed school practice. This lack of detail is not surprising, given that school practices to promote social distancing have not been prominently featured in previous federal guidance to schools, and many state governments use federal guidance to inform their planning efforts [21].

Although we hypothesized that the evidence would be scant, we were surprised to see how few school practices were identified or described. For example, although the Centers for Disease Control and Prevention (CDC) mentions homeroom stay in some of its guidance materials [22], none of the articles, and only two state-level guidance documents we reviewed, mentioned this practice.

Our review of guidance documents identified certain practices that do not appear in the peer-reviewed and grey literature. For example, homeroom stay and visitor restrictions were mentioned in guidance, but did not appear in our peer-reviewed and grey literature review. Furthermore, guidance documents more frequently discussed reducing mixing during transport to and from school.

According to survey data, schools focused their efforts on hand hygiene, respiratory etiquette, and cleaning/disinfecting school buildings, but seldom implemented practices to promote social distancing as a response to the H1N1 pandemic. Nevertheless, published studies that used simulation modeling generally found that practices to promote social distancing could be effective (and less disruptive to society compared to school closure) in curbing disease transmission. However, it is unclear whether these theoretical benefits would be realized following implementation in real-world settings. At the present time, there are only a handful of studies that explore a variety of different practices under a range of different assumptions. As such, it is premature to draw any conclusions about the likely impact of these practices in schools during pandemics.

In the literature and documents we reviewed, school practices were not clearly defined and terms were used inconsistently. This was especially true for school closure. In certain cases, authors used this term to refer to sustained closures (which is out of scope for this review) of weeks or months; however, a one-day “snow day” or class or grade closure were also referred to as “school closure” in select circumstances. To address this issue, we developed a classification system for practices other than sustained school closure and included concrete examples of practices within each defined category. This system, fully characterized in Table 5, can be used going forward to: (1) provide a menu of options to schools selecting among social distancing measures and (2) ensure that educators and policy-makers are speaking a common language regarding school practices to promote social distancing.

Our study has several limitations. First, as described above, terms related to school practices are used inconsistently in the literature and, as such, our search strategy may have inadvertently excluded relevant articles (e.g., articles that refer to school dismissal or school closure but actually cover scenarios of partial closure). Second, we only reviewed publicly available guidance documents targeting local education agencies and/or schools that were posted on the Internet. It is possible that certain states have relevant guidance documents that are not posted and, as such, were not included in this review. It is also possible that, in general, pandemic influenza plans and guidance documents (excluded in this review) could have content relevant to school practices to promote social distancing. To test this possibility, we reviewed eight general pandemic influenza plans from states without school-specific guidance and did not find examples of school social distancing practices other than school closure [23–30]. Despite certain limitations, this is the first review of school practices, other than sustained school closure, and state government guidance on pandemic influenza that we are aware of.

## Conclusions

Our findings suggest that little information is available to schools to develop policies and procedures on social distancing. School leaders and decision-makers can use the findings presented here to understand the range of potential school practices available to them and the evidence supporting their use. Additional research and guidance is needed to assess the feasibility and effectiveness of school practices to promote social distancing to inform federal, state, and local planning efforts going forward.

## Abbreviations

CDC: Centers for disease control and prevention
K-12: Kindergarten through Grade 12
LEA: Local education agency
